# Changes in cross‐sector collaboration between nurse home visitors and community providers in the United States: A panel survey analysis

**DOI:** 10.1111/1475-6773.14242

**Published:** 2023-09-28

**Authors:** Venice Ng Williams, Carol Franco‐Rowe, Michael Knudtson, Gregory Tung, Mandy Allison

**Affiliations:** ^1^ School of Medicine University of Colorado Anschutz Medical Campus, ACCORDS (Adult and Child Center for Outcomes Research and Delivery Science) Aurora Colorado USA; ^2^ Department of Health Systems, Management & Policy Colorado School of Public Health Aurora Colorado USA

**Keywords:** cross‐sector collaboration, home visiting, relational coordination, structural integration, survey research

## Abstract

**Objective:**

Assess changes in cross‐sector collaboration between Nurse‐Family Partnership (NFP) nurse home visitors and community providers in the United States.

**Data Sources and Study Setting:**

We collected primary data via internet‐based surveys of all NFP nursing supervisors in the United States in 2018, 2020, and 2021.

**Study Design:**

We conducted a panel survey to measure changes in cross‐sector collaboration between NFP nurses and 10 provider types in healthcare and social services. We assessed relational coordination using the validated seven item Relational Coordination Scale and structural integration using four items adapted from the Interagency Collaboration Activities Scale. Responses over time were compared using one‐way analysis of variances (ANOVAs) and pairwise *t*‐tests. We used the Kruskal–Wallis rank test to assess differences in collaboration by implementing agency type.

**Data Collection:**

All nursing supervisors from NFP implementing agencies in the United States were eligible for the study. Survey implementation was conducted using Qualtrics and administered to all eligible participants (*N* = 370 [2018], 383 [2020], 414 [2021]). Email reminders were sent every 7–10 days, followed by a final telephone outreach.

**Principal Findings:**

The response rate was 71% in 2018, 83% in 2020, and 74% in 2021. Relational coordination scores were calculated as a mean of the seven items and ranged from 1 to 5 (not at all to completely); integration scores were calculated as a sum of the four items and ranged from 4 to 20, where higher scores indicated greater sharing of resources. Coordination with women's care increased from 2018 to 2020 (*M* = 3.39 vs. 3.57; *p* < 0.01); while coordination (*M* = 3.23 vs. 3.01; *p* < 0.05) and integration (*M* = 6.50 vs. 5.28 vs. 5.43; *p* < 0.01) with parenting programs decreased.

**Conclusions:**

Changes to cross‐sector collaboration varied by provider type, likely due to the delivery of NFP and other services via telehealth during the COVID‐19 pandemic. There is an opportunity to improve cross‐sector collaboration in home visiting to better address family needs.

**What is known on this topic**

- Cross‐sector collaboration can promote health and wellbeing.
- Evidence‐based home visiting programs are proven to improve a range of maternal‐child health outcomes.
- Home visiting programs like Nurse‐Family Partnership are part of the broader systems of care to improve family well‐being.

**What this study adds**

- Cross‐sector collaboration between home visitors and other community service providers varies by provider type and community context.
- Collaboration dynamics further differ by type of agency implementing home visiting, that is, government agency, healthcare, or community‐based organization.
- Coordination between home visiting and women's care providers increased from 2018 to 2021, while coordination and integration with parenting programs decreased.

## 1 INTRODUCTION

Cross‐sector collaboration is the process of partnering between various groups and organizations to collectively focus their expertise and resources on a common goal or problem.^1^, ^2^ Decades of research have identified the need for partnerships across sectors of public health, healthcare, and social services to promote health and well‐being,^3^ particularly among families experiencing adversities. Enhancing collaborative practices across sectors could improve the efficiency and strengthen the overall delivery of home visiting programs, a mechanism of service delivery aimed to meet the needs of families with young children through home‐based services and linkages to community resources.^4^

One of the programs with the strongest evidence for improving maternal‐child health outcomes is Nurse‐Family Partnership (NFP), a national public health program for prenatal and infancy home visiting by nurses. NFP is an evidence‐based home visiting program designed to improve the health and well‐being of first‐time birthing people and their children experiencing economic, social, and/or physical health adversities. The program is based on over 40 years of evidence from three separate randomized clinical trials, with the first trial beginning in the 1970s in Elmira, New York,^5^, ^6^, ^7^ and a fourth recent trial in South Carolina.^8^, ^9^ Since program replication began in the United States in 1996, the program has served over 376,000 families in 774 counties among 40 states and the US Virgin Islands.^10^ The program has three major aims: (1) to improve pregnancy outcomes, (2) to improve child health and development, and (3) to increase families' economic self‐sufficiency. Trained nurses visit eligible birthing people early in their pregnancy through child age two, providing support and education, as well as linking families to needed community services. NFP nurses follow protocols that are grounded in theories of developmental epidemiology, human attachment, human ecology, and self‐efficacy and tailor the intervention to meet families' needs.^11^

Increasing home visiting nurses' ability to address maternal and child health risks through integrated approaches and cross‐sector partnerships could help to ensure that families receive needed services and continuity of care.^12^ However, difficulties exist in coordinating services across providers in different sectors.^13^ The ongoing global pandemic of the coronavirus disease 2019 (COVID‐19) caused by severe acute respiratory syndrome coronavirus 2 (SARS‐CoV‐2) has made these challenges more urgent, and the implications of services not meeting the needs for family health have become more evident.

Our conceptualization of cross‐sector collaboration is driven by relational coordination theory coupled with structural integration. Relational coordination theory posits that relationships are characterized by shared goals, shared knowledge, and mutual respect that support frequent, timely, accurate, problem‐solving communication to enable partners to effectively coordinate their work.^14^ Structural integration supports relational coordination based on the design of organizational structures. Coordination is more reliable when opportunities to work together are built into organizational structures such as shared information systems, protocols or agreements, space, and accountability or rewards (e.g., financial alignment or incentives).^15^, ^16^

Previous research has examined service coordination with community resources by home visiting programs, including screening, referrals, linkages, and follow‐up with referrals. One study of a national sample of home visiting sites found that sites varied in their coordination activities, with screening and referrals happening more frequently than linkages and follow‐up.^17^ Qualitative studies have identified facilitators and barriers to service coordination and cross‐sector collaboration including shared mission and goals^18^; integrated structures, personal relationships^19^; availability and accessibility of resources^20^; and the role of the warm hand‐offs.^21^ Few studies have examined if better coordination and collaboration improve family outcomes. One study found that better coordination for maternal depression improved access to services and depressive symptoms^22^; our previous work found that strong collaboration improves participant retention in nurse home visiting.^23^

However, current evidence is lacking regarding the number and breadth of cross‐sector partnerships between community‐based organizations (CBOs) and providers who address the needs of families experiencing adversity. Further, no research has measured changes in partnerships between home visitors and other service providers over time. The purpose of this study was to assess changes in cross‐sector collaboration between NFP nurse home visitors and community providers in the United States using a panel survey. We assessed how collaboration measures with all providers vary over time. Our study explores collaboration beyond service coordination to capture shared communication, relationships, and structures. Specifically, we hypothesized that coordination and integration with healthcare providers (i.e., women's care, pediatrics care, mental health) would increase over time, given the NFP's program's strategic promotion of model integration with healthcare delivery and payer systems over the past few years. For example, NFP has explored the use of healthcare entities in implementing the program, reimbursement/funding of the program through Medicaid, and access to/charting in clients' electronic medical records.

Although not originally intended, our survey was implemented during the COVID‐19 pandemic, which offered an opportunity to examine cross‐sector collaboration changes during this unprecedented time. Note that we use the term women's care providers in this manuscript and in our survey, as this was the commonly understood verbiage among our participants to describe perinatal obstetrical and gynecological services for birthing people. We acknowledge that not all birthing people identify as women.

## 2 METHODS

### 2.1 Study design and participants

We conducted a web‐based survey with NFP teams from implementing agencies across the United States actively operating the NFP program at three time points: in the fall of 2018, 2020, and 2021. We used a contact list from the NFP National Service Office, the nonprofit agency responsible for overseeing the implementation of NFP in the United States, to recruit nurse supervisors to participate on behalf of eligible teams. If an implementing agency did not have an identified nurse supervisor on the contact list (e.g., the position was vacant), we invited the administrator or nurse home visitor serving as interim supervisor to participate. NFP nurse supervisors were invited to participate in the survey via email through Qualtrics. The study received ethical approval from the researchers' local Institutional Review Board.

### 2.2 Variables and definitions

#### 2.2.1 Relational coordination

The validated Relational Coordination Scale is based on the relational coordination theory for understanding the relational dynamics of coordinating work. It measures high‐quality communication as a function of frequency, timeliness, accuracy, and problem‐solving; and high‐quality relationships based upon shared goals, shared knowledge, and mutual respect.^15^ This scale uses seven items with five response options ranging from never/nothing/not at all to constantly/completely (coded numerically from 1 to 5).

#### 2.2.2 Structural integration

The Structural Integration measure uses four items adapted from the 17‐item Interagency Collaboration Activities Scale to capture other collaborative activities of an organization from a structural perspective. It measures the degree of shared resources, in terms of shared facility space, shared data or information systems, shared policies or written agreements, and shared funding or financial alignment. Response options ranged from not at all to very much (coded numerically from 1 to 5).

For both scales, nurse supervisors were asked about their perceptions of relational coordination and structural integration with nine provider types in 2018, including four healthcare (women's care, pediatric care, mental health, substance use treatment) and five social services (child welfare, Special Supplemental Nutrition Program for Women, Infants and Children—WIC, parenting programs, housing resources, and early intervention); other home visiting programs were added as a tenth provider type in 2020 and 2021 based on feedback from our project's Advisory Board, as NFP clients are usually able to participate in more than one home visiting service. The validity of the two scales in the home visiting setting is described elsewhere.^24^ See Appendix for scale verbiage.

#### 2.2.3 Other data sources

We also used NFP program implementation data available through the NFP Data Warehouse for additional analyses. We included nurse supervisor and agency‐level variables. Nurse supervisor variables were nurse tenure (years employed by NFP), race, ethnicity, and highest education level. Agency‐level variables were program tenure (years implementing NFP), agency type (organization type implementing NFP), and state.

Agency type is categorized as a government agency, healthcare, CBO, or educational institution. A government agency is defined as any organization that is a government entity or has a governing body, typically a local, regional or state health department, human/social services department, or health and human services department. A healthcare agency is defined as any organization that is associated with healthcare payment or healthcare delivery, such as a managed care organization, health system or hospital, clinic or visiting nurse service. A CBO is a nonprofit organization that works to meet community needs, including foundations and faith‐based organizations. Finally, educational institutions are defined as any private or public institution that delivers education, such as a high school district, and higher education like a community college or university.

### 2.3 Data collection

A web‐based survey was administered to all eligible teams: *N* = 370 in 2018, *N* = 383 in 2020, and *N* = 414 in 2021. The same teams invited in 2018 were invited again in 2020 or 2021, unless they were no longer actively implementing the NFP program. New teams that began NFP implementation after 2018 were added to subsequent waves of data collection in 2020 and 2021. Participants could respond to the survey via a personal link in their email invitation. Four email reminders were sent every seven to 10 days, followed by a final telephone outreach, resulting in five varied contacts as suggested by the Dillman method in accordance with best practices in survey research.^25^ The survey was open for 6 weeks. There was no monetary incentive offered in 2018. In 2020, we provided an advance incentive of donating $2000 to Cribs for Kids on behalf of the NFP program, in honor of October being Safe Sleep Awareness Month. In 2020 and 2021, we provided a secondary incentive of $50 gift certificates to Amazon or the NFP store to four randomly selected NFP teams who completed the survey to purchase items for their nursing team or clients.

### 2.4 Data analysis

Responses by provider type to each of the seven relational coordination items were averaged to produce a relational coordination score that provides a measure of the level of coordination with a specific provider type. Responses to each dimension of relational coordination were also averaged across provider types. In other words, there is a relational coordination score for each of the nine provider types (e.g., relational coordination with women's care providers) and for each dimension (e.g., frequency of communication).

Similarly, responses by provider type to each of the four structural integration items were added together to produce an integration score for the level of shared structures with a specific provider type. Responses to each dimension of structural integration were also averaged across provider types. Index scores were further created as a mean of all scores across provider types, producing a relational coordination index score and a structural integration score to characterize overall coordination and integration across all provider types. For each provider type, respondents who answered four or more of the seven relational coordination items were included in the analysis to maximize the sample size after examination of the missing data. For each provider type, respondents who answered three or more of the four structural integration items were included in the analysis, again to maximize the sample size.

Survey data were matched to NFP implementation data using the respondent's agency identification number, email address, and first and last names. Data were analyzed with descriptive statistics; responses over time were compared using one‐way analysis of variances (ANOVAs) and pairwise *t*‐tests with a Bonferroni correction. We used the Kruskal–Wallis rank test to assess differences in 2021 collaboration measures by agency type. To ensure we did not have nonresponse bias, we compared the characteristics of respondents and non‐respondents using two‐sample *t*‐tests or Chi‐square tests.

## 3 RESULTS

The response rate was 71% (263/370) in 2018, 83% (316/383) in 2020, and 74% (307/414) in 2021. The majority of participants were nursing supervisors (95% in 2020, 95% in 2020, and 93% in 2021), primarily employed by government agencies like public health departments (52% in 2020, 52% in 2020, and 50% in 2021; see Table 1 for characteristics of the survey respondents). We did not find any differences between respondents and non‐respondents based on nurse or agency‐level characteristics in any year that the survey was conducted, except for differences in supervisor education level in 2020 (*χ* ^2^ = 8.32, *p* < 0.05; see Table 2 for results of tests for differences).

**TABLE 1:** Characteristics of survey respondents.

	2018	2020	2021
Survey respondents, *N*	263	316	307
Nurse supervisor, *N* (%)	250 (95%)	300 (95%)	285 (93%)
Other: Nurse home visitor, administrator, *N* (%)	13 (5%)	16 (5%)	22 (7%)
Localities represented			
Teams, *N*	257	301	298
Sites, *N*	199	229	227
States and territories, *N*	39	39	42
Agency type			
Government agency, *N* (%)	137 (52%)	162 (52%)	153 (50%)
Healthcare, *N* (%)	50 (19%)	70 (22%)	67 (21%)
Community‐based organization, *N* (%)	68 (26%)	74 (23%)	76 (25%)
Educational institution/other, *N* (%)	8 (3%)	10 (3%)	11 (4%)

**TABLE 2:** Tests for differences between survey respondents and non‐respondents.

	2018			2020			2021		
	Respondent	Non‐respondent	Test statistic	Respondent	Non‐respondent	Test statistic	Respondent	Non‐respondent	Test statistic
Program tenure, years (*N*)	11.54 (232)	11.81 (106)	*t* = 0.44	12.35 (310)	13.10 (64)	*t* = 0.71	13.33 (295)	13.67 (90)	*t* = 0.44
Agency type, *N* (%)									
Government agency	130 (50%)	71 (65%)	*χ* ^2^ = 7.10	164 (52%)	46 (68%)	*χ* ^2^ = 5.94	153 (50%)	54 (58%)	*χ* ^2^ = 2.92
Healthcare	51 (20%)	18 (16%)		74 (23%)	11 (16%)		67 (22%)	16 (18%)	
Community‐based organization	71 (27%)	19 (17%)		70 (22%)	10 (15%)		76 (25%)	19 (20%)	
Educational Institution/Other	8 (3%)	2 (2%)		10 (3%)	1 (1%)		11 (3%)	4 (4%)	
Nurse tenure, years (*N*)	N/A^a^	N/A^a^	N/A	6.29 (276)	6.57 (54)	*t* = 0.39	7.81 (304)	7.02 (93)	*t* = 0.44
Nurse race, *N* (%)	N/A^a^	N/A^a^	N/A						
White				185 (62%)	35 (57%)	*χ* ^2^ = 0.93	173 (63%)	48 (65%)	*χ* ^2^ = 1.42
African‐American/Black				31 (10%)	8 (13%)		34 (12%)	9 (12%)	
Other race				6 (2%)	2 (3%)		5 (2%)	0 (0%)	
Mixed race				4 (1%)	1 (2%)		3 (1%)	1 (1%)	
Declined				75 (25%)	15 (25%)		60 (22%)	16 (22%)	
Nurse ethnicity, *N* (%)	N/A^a^	N/A^a^	N/A						
Hispanic				16 (5%)	7 (10%)	*χ* ^2^ = 2.90	16 (6%)	8 (11%)	*χ* ^2^ = 5.69
Non‐Hispanic				181 (57%)	35 (52%)		169 (61%)	35 (46%)	
Unknown				121 (38%)	26 (38%)		92 (33%)	33 (43%)	
Nurse highest education, *N* (%)	N/A^a^	N/A^a^	N/A						
Diploma/associates degree				6 (2%)	3 (5%)	*χ* ^2^ = 8.32*	5 (3%)	2 (2%)	*χ* ^2^ = 0.37
Bachelor's degree				225 (73%)	32 (55%)		198 (73%)	55 (72%)	
Master's degree or higher				76 (25%)	23 (40%)		73 (24%)	18 (26%)	

^a^ Data not available.

* *p* < 0.05.

### 3.1 Main results

Descriptive results for relational coordination and structural integration scores by provider type and year are shown in Tables 3 and 4 respectively. Index scores are calculated as a mean of all scores across providers (relational coordination and structural integration separately). Relational coordination scores are calculated as a mean of the seven items and ranged from 1 to 5 (not at all to completely). NFP nurse supervisors reported moderate relational coordination among all providers (*M* = 3.21 in 2018, 3.21 in 2020, 3.23 in 2021). By provider type, the highest reported coordination was with WIC (supplemental nutrition; *M =* 3.77 in 2018, 3.68 in 2020, 3.67 in 2021) and women's care providers (*M =* 3.39 in 2018, 3.57 in 2020, 3.51 in 2021). The lowest reported coordination was with housing resources (*M =* 2.55 in 2018, 2.50 in 2020, 2.61 in 2021) and substance use treatment providers (*M =* 2.74 in 2018, 2.76 in 2020, 2.88 in 2021).

**TABLE 3:** Relational coordination scores by year and analysis of variance (ANOVA) results.

	2018			2020			2021			ANOVA results			
	*N*	Mean	SD	*N*	Mean	SD	*N*	Mean	SD	All years	2018 versus 2020	2018 versus 2021	2020 versus 2021
Relational coordination index score across all providers	236	3.21	0.62	296	3.21	0.54	278	3.23	0.78	*F*_*2*,808_ = 0.36	*t* = −0.01	*t* = 0.03	*t* = 0.04
Relational coordination score by provider type													
WIC^a^ (supplemental nutrition)	235	3.77	0.90	296	3.68	0.82	276	3.67	0.83	*F*_*2*,805_ = 1.46	*t* = −0.11	*t* = −0.11	*t* = 0.00
Women's care	236	3.39	0.79	300	3.57	0.73	277	3.51	0.75	*F*_*2*,811_ = 3.67*	*t* = 0.18*	*t* = 0.12	*t* = −0.06
Early intervention	233	3.44	0.90	290	3.36	0.80	271	3.39	0.78	*F*_*2*,792_ = 1.17	*t* = −0.11	*t* = −0.06	*t* = 0.06
Other home‐visiting service^b^	‐	‐	‐	284	3.28	0.84	260	3.39	0.79	*F*_*1*,541_ = 2.78	N/A	N/A	*t* = 0.12
Mental health	232	3.24	0.83	297	3.25	0.73	276	3.31	0.77	*F*_*2*,803_ = 0.77	*t* = 0.00	*t* = 0.07	*t* = 0.07
Child welfare	234	3.28	0.73	294	3.26	0.68	275	3.21	0.72	*F*_*2*,801_ = 0.54	*t* = −0.04	*t* = −0.07	*t* = −0.03
Pediatric care	234	3.13	0.82	294	3.14	0.76	276	3.16	0.82	*F*_*2*,802_ = 0.09	*t* = 0.00	*t* = 0.03	*t* = 0.03
Parenting programs	222	3.23	0.95	262	3.01	0.85	256	3.14	0.78	*F*_*2*,737_ = 4.31*	*t* = −0.22*	*t* = −0.08	*t* = 0.14
Substance use treatment	219	2.74	0.89	286	2.76	0.85	260	2.88	0.87	*F*_*2*,764_ = 2.59	*t* = 0.01	*t* = 0.17	*t* = 0.15
Housing resources	225	2.55	0.93	283	2.50	0.84	260	2.61	0.86	*F*_*2*,767_ = 1.64	*t* = −0.07	*t* = 0.07	*t* = 0.14
Relational coordination domains across all provider types													
Shared goals	227	3.55	0.85	289	3.60	0.79	271	3.62	0.79	*F*_*2*,785_ = 0.46	*t* = 0.04	*t* = 0.07	*t* = 0.03
Mutual respect	226	3.54	0.76	283	3.55	0.75	268	3.59	0.76	*F*_*2*,775_ = 0.57	*t* = 0.01	*t* = 0.07	*t* = 0.06
Accuracy of communication	230	3.40	0.96	279	3.37	0.94	265	3.39	0.95	*F*_*2*,772_ = 0.08	*t* = −0.03	*t* = −0.00	*t* = 0.03
Shared knowledge	229	3.20	0.66	293	3.24	0.63	273	3.23	0.65	*F*_*2*,793_ = 0.26	*t* = 0.03	*t* = 0.04	*t* = 0.01
Problem solving	229	3.13	0.79	283	3.07	0.80	269	3.11	0.82	*F*_*2*,777_ = 0.62	*t* = −0.08	*t* = −0.03	*t* = 0.05
Timeliness of communication	231	3.06	0.77	287	3.00	0.77	272	3.09	0.80	*F*_*2*,788_ = 1.12	*t* = −0.06	*t* = 0.03	*t* = 0.10
Frequency of communication	238	2.87	0.65	298	2.75	0.59	278	2.84	0.66	*F*_*2*,812_ = 3.79*	*t* = −0.14*	*t* = −0.03	*t* = 0.11

^a^ Special supplemental nutrition program for Women, Infants, and Children (WIC).

^b^ Data not available.

*Adjusted *p* < 0.05, **p < 0.01, ***p < 0.001.

**TABLE 4:** Structural integration scores by year and analysis of variance (ANOVA) results.

	2018			2020			2021			ANOVA results			
	*N*	Mean	SD	*N*	Mean	SD	*N*	Mean	SD	All years	2018 versus 2020	2018 versus 2021	2020 versus 2021
Structural integration index score across all providers	225	6.07	1.61	297	6.02	1.8	273	6.07	1.61	*F*_*2*,792_ = 0.19	*t* = −0.08	*t* = −0.01	*t* = 0.07
Structural integration score by provider type													
Other home‐visiting service^a^	‐	‐	‐	295	8.20	4.91	271	8.21	5.05	*F*_*1*,563_ = 0.02	N/A	N/A	*t* = 0.07
WIC^b^ (supplemental nutrition)	218	8.03	4.17	295	7.75	4.43	273	7.68	4.27	*F*_*2*,786_ = 0.59	*t* = −0.33	*t* = −0.40	*t* = −0.07
Mental health	223	7.06	3.86	293	6.29	3.84	273	6.75	4.07	*F*_*2*,788_ = 2.47	*t* = −0.75	*t* = −0.28	*t* = 0.48
Women's care	225	6.60	3.56	296	6.69	4.15	273	6.50	3.63	*F*_*2*,793_ = 0.17	*t* = 0.02	*t* = −0.15	*t* = −0.17
Early intervention	219	5.70	3.25	296	5.64	3.07	272	5.78	3.24	*F*_*2*,786_ = 0.33	*t* = −0.16	*t* = 0.04	*t* = 0.21
Pediatric care	224	5.92	3.31	295	5.65	3.32	273	5.68	3.15	*F*_*2*,791_ = 0.70	*t* = −0.33	*t* = −0.26	*t* = 0.07
Parenting programs	218	6.50	3.65	294	5.28	2.9	272	5.43	2.87	*F*_*2*,783_ = 10.00***	*t* = −1.16***	*t* = −1.02***	*t* = 0.14
Child welfare	217	5.28	2.44	297	5.19	2.26	273	5.24	2.01	*F*_*2*,786_ = 0.22	*t* = −0.12	*t* = −0.03	*t* = 0.09
Substance use treatment	222	5.07	2.42	294	5.17	2.87	273	4.97	2.27	*F*_*2*,788_ = 0.35	*t* = 0.08	*t* = −0.10	*t* = −0.18
Housing resources	218	4.44	1.39	296	4.44	1.33	270	4.44	1.51	*F*_*2*,783_ = 0.00	*t* = −0.00	*t* = 0.01	*t* = 0.01
Structural integration domains across all provider types													
Shared space	225	1.68	0.59	297	1.65	0.59	273	1.70	0.55	*F*_*2*,794_ = 0.59	*t* = −0.04	*t* = 0.01	*t* = 0.05
Shared policies	225	1.65	0.77	297	1.70	0.70	273	1.66	0.66	*F*_*2*,794_ = 0.28	*t* = 0.04	*t* = 0.01	*t* = −0.03
Shared data	225	1.44	0.55	297	1.38	0.52	273	1.41	0.51	*F*_*2*,794_ = 0.92	*t* = −0.06	*t* = −0.03	*t* = 0.04
Shared funding	225	1.31	0.41	297	1.31	0.43	273	1.32	0.39	*F*_*2*,794_ = 0.12	*t* = 0.00	*t* = 0.01	*t* = 0.01

^a^ Data not available.

^b^ Special supplemental nutrition program for Women, Infants, and Children (WIC).

*Adjusted *p* < 0.05, **p < 0.01, ***p < 0.00.

Structural integration scores are a sum of the four items by provider type, then averaged across respondents, and ranged from 4 to 20, where higher scores indicate greater sharing of resources. NFP nurse supervisors reported little integration among all providers (*M* = 6.07 in 2018, 6.02 in 2020, 6.07 in 2021). The greatest reported integration was with other home visiting services (*M* = 8.20 in 2020, 8.21 in 2021) and WIC (*M* = 8.03 in 2018, 7.75 in 2020, 7.68 in 2021). Similar to coordination, the lowest reported integration was with housing resources (*M* = 4.44 in 2018, 4.44 in 2020, 4.44 in 2021) and substance use treatment providers (*M* = 5.07 in 2018, 5.17 in 2020, 4.97 in 2021).

Results for relational coordination and structural integration domains across provider types are shown by year in Tables 3 and 4 respectively. The highest‐rated dimension of relational coordination across all providers was shared goals (*M =* 3.55 in 2018, 3.60 in 2020, 3.62 in 2021), and frequent communication (*M =* 2.87 in 2018, 2.75 in 2020, 2.84 in 2021) was the least endorsed. Dimensions of structural integration across providers ranged from 1 through 5, where physical space was rated the highest (*M* = 1.68 in 2018, 1.65 in 2020, 1.70 in 2021) and shared funding the lowest (*M* = 1.31 in 2018, 1.31 in 2020, 1.32 in 2021).

In terms of collaboration changes over time, we found differences in relational coordination and structural integration among a few provider types. Coordination with women's care increased from 2018 to 2020 (*M* = 3.39 vs. 3.57; *t* = 0.18; Bonferroni‐corrected *p* = 0.02); while coordination with parenting programs decreased from 2018 to 2020 (*M* = 3.23 vs. 3.01; *t* = −0.22; Bonferroni‐corrected *p* = 0.01). Changes in coordination with other home visiting services neared significance and increased from 2020 to 2021 (*M* = 3.28 vs. 3.39; *t* = 0.12; Bonferroni‐corrected *p* = 0.09). Integration with parenting programs decreased from 2018 to 2020 and 2021 (*M* = 6.50 vs. 5.28 vs. 5.43; *t* = −1.16 and − 1.02 comparing 2018 to 2020 and 2018 to 2021; Bonferroni‐corrected *p* = 0.00 for both comparisons), but not from 2020 to 2021 (*M* = 5.64; *t* = 0.12; Bonferroni‐corrected *p* = 0.09). Changes in integration with mental health providers neared significance and decreased from 2018 to 2020 (*M* = 7.06 vs. 6.29; *t* = −0.75; Bonferroni‐corrected *p* = 0.09). Coordination and integration with all other providers did not change over time (see Tables 3 and 4). With regards to collaboration domains across provider types, the frequency of communication was reported to decrease from 2018 to 2020 (*M* = 2.87 vs. 2.75; *t* = 0.14; Bonferroni‐corrected *p* = 0.03). Changes in reporting of all other collaboration domains did not change significantly over time (see Tables 3 and 4).

### 3.2 Collaboration differences by agency type

The results of the Kruskal–Wallis Chi‐squared test were significant, suggesting differences in the degree of collaboration by NFP implementing agency. Relational coordination with women's care (*χ* ^2^ = 16.56, *p* < 0.01), pediatrics care (*χ* ^2^ = 10.70, *p* < 0.05), and supplemental nutrition (*χ* ^2^ = 35.38, *p* < 0.01) differed by agency type (see Table 5). Specifically, relational coordination with women's care among NFP sites implemented by healthcare agencies was the strongest (*M* = 3.78) compared to government (*M* = 3.49), CBOs (*M* = 3.41), and educational institutions (*M* = 2.78). Similarly, coordination with pediatric care among healthcare NFP sites was the strongest among agency types (*M* = 3.35) compared to government (*M* = 3.16), CBOs (*M* = 3.04), and educational institutions (*M* = 2.59). Coordination with WIC was strongest among NFP sites implemented by government agencies (*M* = 3.93) compared to healthcare agencies (*M* = 3.60), CBOs (*M* = 3.32) and educational institutions (*M* = 2.74).

**TABLE 5:** Collaboration differences by agency type.

	Government agency, mean (SE), *N*	Healthcare, mean (SE), *N*	Community‐based organization, mean (SE), *N*	Education, mean (SE), *N*	Test statistic (*χ* ^2^)
Relational coordination with…					
WIC^a^ (supplemental nutrition)	3.93 (0.07), 137	3.60 (0.07), 60	3.32 (0.10), 70	2.74 (0.31), 7	*χ* ^2^ = 35.38*** *p* = 0.00
Women's care	3.49 (0.06), 136	3.78 (0.06), 62	3.41 (0.09), 70	2.78 (0.28), 7	*χ* ^2^ = 16.56*** *p* = 0.00
Early intervention	3.42 (0.07), 134	3.46 (0.07), 60	3.30 (0.09), 68	3.04 (0.29), 7	*χ* ^2^ = 4.14 *p >* 0.10
Other home‐visiting service	3.37 (0.07), 127	3.54 (0.07), 58	3.35 (0.10), 66	2.94 (0.30), 7	*χ* ^2^ = 5.35 *p >* 0.10
Mental health	3.30 (0.07), 135	3.30 (0.07), 62	3.35 (0.09), 70	2.96 (0.29), 7	*χ* ^2^ = 1.52 *p >* 0.10
Child welfare	3.22 (0.06), 136	3.30 (0.06), 60	3.18 (0.09), 70	2.69 (0.27), 7	*χ* ^2^ = 5.87 *p >* 0.10
Pediatric care	3.16 (0.07), 135	3.35 (0.07), 62	3.04 (0.10), 70	2.59 (0.31), 7	*χ* ^2^ = 10.70** *p* = 0.01
Parenting programs	3.13 (0.07), 124	3.28 (0.07), 59	3.06 (0.10), 64	2.92 (0.29), 7	*χ* ^2^ = 3.95 *p >* 0.10
Substance use treatment	2.90 (0.08), 127	2.96 (0.08), 57	2.83 (0.11), 67	2.39 (0.33), 7	*χ* ^2^ = 4.06 *p* > 0.10
Housing	2.56 (0.08), 129	2.72 (0.08), 55	2.64 (0.11), 67	2.29 (0.33), 7	*χ* ^2^ = 2.65 *p >* 0.10
Structural integration with…					
Other home‐visiting service	7.76 (0.44), 134	7.90 (0.44), 62	9.53 (0.62), 66	8.29 (1.91), 7	*χ* ^2^ = 3.56 *p* > 0.10
WIC^a^ (supplemental nutrition)	9.74 (0.37), 134	5.71 (0.37), 63	5.64 (0.52), 67	4.29 (1.61), 7	*χ* ^2^ = 70.93*** *p* = 0.00
Mental health	6.16 (0.36), 134	6.49 (0.36), 63	8.19 (0.51), 67	6.00 (1.58), 7	*χ* ^2^ = 7.52 *p* > 0.05
Women's care	6.01 (0.31), 134	8.24 (0.31), 63	5.72 (0.43), 67	5.86 (1.34), 7	*χ* ^2^ = 19.23*** *p* = 0.00
Early intervention	5.43 (0.28), 134	6.35 (0.28), 63	6.14 (0.40), 66	4.57 (1.23), 7	*χ* ^2^ = 2.52 *p >* 0.10
Pediatric care	5.22 (0.27), 134	6.97 (0.27), 63	5.40 (0.38), 67	4.43 (1.18), 7	*χ* ^2^ = 8.96* *p* = 0.03
Parenting programs	5.16 (0.25), 134	5.54 (0.25), 63	5.86 (0.35), 66	5.14 (1.08), 7	*χ* ^2^ = 0.91 *p* > 0.10
Child welfare	5.45 (0.17), 134	5.02 (0.17), 63	5.19 (0.25), 67	4.00 (0.76), 7	*χ* ^2^ = 8.61* *p* = 0.04
Substance use treatment	4.73 (0.20), 134	5.60 (0.20), 63	4.88 (0.28), 67	4.14 (0.86), 7	*χ* ^2^ = 3.09 *p >* 0.10
Housing resources	4.26, 134	4.61, 62	4.57, 65	5.14, 7	*χ* ^2^ = 4.11 *p >* 0.10

^a^ Special supplemental nutrition program Women, Infants, and Children (WIC).

* *p* < 0.05;

** *p* < 0.01;

*** *p* < 0.001.

We found similar results for measures of structural integration with the same provider types (women's care, pediatric care, and WIC), but also with child welfare. Structural integration with women's care (*χ* ^2^ = 19.23, *p* < 0.01), pediatrics care (*χ* ^2^ = 8.96, *p* < 0.05), WIC (*χ* ^2^ = 70.93, *p* < 0.01), and child welfare (*χ* ^2^ = 8.61, *p* < 0.05) differed by agency type (see Table 5). Like with relational coordination, structural integration with women's care among NFP sites implemented by healthcare agencies was the greatest (*M* = 8.24) compared to government (*M* = 6.01), education (*M* = 5.86), and CBOs (*M* = 5.72). Similarly, integration with pediatric care among healthcare NFP sites was the greatest among agency types (*M* = 6.97) compared to CBOs (*M* = 5.40), government (*M* = 5.22), and education (*M* = 4.43). Integration with WIC among NFP sites implemented by government agencies was the greatest (*M* = 9.74) compared to healthcare (*M* = 5.71), CBOs (*M* = 5.64), and education (*M* = 4.29). Similarly, integration with child welfare among NFP sites implemented by government agencies was the greatest (*M* = 5.45) compared to CBOs (*M* = 5.19), healthcare (*M* = 5.02), and education (*M* = 4.00).

## 4 DISCUSSION

This study aimed to assess changes in cross‐sector collaboration, as measured by relational coordination and structural integration, over time between NFP nurses and 10 different healthcare and social service provider types. These included four healthcare provider types (women's care, pediatric care, mental health, and substance use treatment) and six social service provider types (other home visiting programs, child welfare, WIC, parenting programs, housing resources, and early intervention). Specifically, we aimed to test whether collaboration with healthcare providers (women's and pediatric care) would increase over time. While it is widely believed that cross‐sector collaboration is key to meeting the needs and improving outcomes for those facing the greatest adversity, the exact nature of effective collaborative relationships is still not well understood and studies examining the association of measures of collaboration and health outcomes have shown mixed results.^20^, ^21^, ^22^ This study used more nuanced measures of collaboration over time that recognize the multiple dimensions of collaboration and that collaboration dynamics are specific to different provider types and types of implementing agencies.

Overall relational coordination scores for NFP nurses ranged from 2.50 to 3.77, with an average of 3.22 which equates to occasional/moderate coordination. Compared to other studies, NFP nurses coordinate with other providers to a similar degree to nurses in home health (scores range from 3.41 to 4.20),^26^, ^27^ nurses in acute care (scores range from 2.98 to 4.19),^28^, ^29^ and public health department staff (scores range from 2.20 to 3.97).^30^ Existing literature suggests that the design of organizational structures strengthens relational coordination‐like practices (i.e., hiring and training for teamwork, shared accountability and reward structures) and coordinating mechanisms (i.e., shared standardized work protocols, shared information systems, regular meetings).^14^

We saw generally less collaboration between NFP and providers in housing resources and substance use treatment; though both sectors represent critical needs for some families. Our prior qualitative inquiries have found that NFP nurses rarely share client information with housing service providers.^19^ Despite various housing resources available for their clients, the demand for affordable housing far exceeded what was available; limiting opportunities for NFP and housing providers to coordinate. With regards to collaboration with substance use treatment providers, the community variation in the availability of treatment and support for pregnant and parenting people along with low proportions of NFP clients with substance use disorder may contribute to the lower collaboration scores reported.

Our findings suggest NFP collaboration with providers working in other sectors changed little during our study timeframe. The small changes to collaboration over time varied by provider type, and overall collaboration measures differed by type of NFP implementing agency. We found that coordination with women's care providers increased from 2018 to 2020, while integration with women's care, as well as integration and coordination with pediatric care did not. These results do not fully align with our hypothesis that coordination and integration with healthcare providers (i.e., women's care, pediatrics care, mental health) would increase over time. Despite the NFP's national strategic promotion of model integration with healthcare systems, it is possible that the COVID‐19 pandemic halted these efforts given that many health systems were overwhelmed during this time.

We also saw an increase in the frequency of communication from 2018 to 2020 among all provider types. In response to the COVID‐19 pandemic, HRSA released guidance to encourage home visiting service delivery via telehealth and recommended home visitors to partner with healthcare providers to ensure that pregnant and postpartum women have access to prenatal and postnatal care.^31^ The increase in NFP coordination with women's care providers may relate to the delivery of the program via telehealth rather than the traditional in‐person format during the pandemic.^32^ A recent survey of multiple home visiting models found that home visitors communicated most regularly with pediatric, prenatal, and adult primary care providers to discuss specific health‐related concerns, inform screening results, and to clarify medical advice given to clients.^33^ Therefore, it is likely that NFP nurses felt a greater need to communicate and coordinate with women's care providers during the pandemic because they were not physically visiting their clients in 2020. As NFP nurses were unable to conduct physical assessments with their clients, NFP nurses may have needed to coordinate with women's care providers to ensure that their clients' physical health in the perinatal period was being assessed and monitored. However, we did not see this same dynamic in terms of coordination with pediatric care providers among our survey respondents. This may be due to NFP nurses having different relationships with women's care versus pediatric care providers; many women's care providers refer their patients to NFP and are familiar with what NFP nurses offer to families, which are factors that facilitate care coordination.^19^

We also found that relational coordination and structural integration with parenting programs decreased from 2018 to 2020 and from 2018 to 2021, but not from 2020 to 2021. As we added “other home visiting service” as a provider type in the survey conducted in 2020 and continued to include it in 2021, it is likely that “parenting programs” had previously captured the collaboration dynamic of “other home visiting services” and offering “other home visiting service” as a separate provider type provided a more accurate reflection of NFP nurses' coordination and integration with parenting programs. At the same time, the COVID‐19 pandemic led to the shutdown or diversion of certain non‐essential services, including parenting programs. If these programs were not being implemented and NFP clients were not participating in them, NFP nurses would not have needed to coordinate with these providers during this time period.

In addition to changes in collaboration over time, our findings suggest differences in collaboration by NFP implementing agency type. NFP sites implemented by healthcare agencies had stronger relational coordination and integration with women's care and pediatric care providers compared to NFP sites implemented by other agency types. If a healthcare entity is implementing NFP, the NFP program likely shares physical space (i.e., a building or campus), data or information systems (i.e., electronic medical record), and policies with women's and pediatric care providers (i.e., informed consent and patient information compliance). Sharing this infrastructure including co‐location, common communication channels, and formal policies facilitate relational coordination with these types of healthcare providers.^15^, ^16^ Then NFP nurses and healthcare providers are more likely to be aware of one another's program offerings (shared knowledge), and have the same organizational mission and vision (shared goals), which contribute to respecting one another's role and profession (mutual respect). These factors then facilitate high‐quality communication that is frequent, accurate, timely and/or problem‐solving in nature.^34^ Our findings are consistent with recent research suggesting that implementing agencies that are healthcare organizations have more regular communication with the mother's and child's healthcare providers and that home visiting programs with a memorandum of understanding with a healthcare provider are significantly more likely to have regular communication with those providers.^33^

NFP sites that were implemented by government agencies had stronger relational coordination and integration with WIC providers compared to NFP sites implemented by other agency types. Many government agencies that implement NFP are local public health departments, which often house WIC services.^35^ From an infrastructure standpoint, NFP and WIC often share the same building (even floor), abide by the same policies, have access to one another's data systems, and may even have the same funding sources which are all domains of structural integration.^24^ In these situations, where the two programs are operated by the same organization, NFP nurses and WIC providers, are more likely to have high‐quality relationships and high‐quality communications (relational coordination)^34^ due to the examples provided above.

Similarly, NFP sites that were implemented by government agencies were more integrated with child welfare than NFP sites implemented by other agency types. In many communities, local public health departments are often integrated with social services; these organizations are usually called departments of human services, social services, or health and human services. Similar to WIC, child welfare is typically operated out of these local departments and is designed to promote the well‐being of children by ensuring safety, achieving permanency, and strengthening families through child protective services interventions.^36^ When NFP nurses are housed in the same organization that operates child welfare, they have the same infrastructure and often access to the same resources (structural integration)^24^ which facilitate high‐quality relationships and high‐quality communication (relational coordination).^37^

### 4.1 Strengths and limitations

This study is the first to document changes in cross‐sector collaboration over time in NFP and broadly within the context of home visiting. We had a high response rate among nursing supervisors representing 78% of NFP implementing agencies. There were no significant differences in most nurse supervisor and all agency‐level characteristics between respondents and non‐respondents. However, there is still potential for response bias based upon other unmeasured factors. Further, NFP nurses are motivated to partake in research that benefits the program, so there is potential for social desirability bias. However, given that we found variation in the degree of collaboration by provider type across NFP sites, we believe there is limited response bias. This study was conducted within the context of the NFP program, which should be taken into consideration when assessing the generalizability of the findings to other community‐based interventions focused on improving maternal‐child health. The ANOVA post‐hoc tests are adjusted for tests within each provider type and not for multiple comparisons across provider types; it is possible that the few significant findings may be due to chance alone.

### 4.2 Implications for practice, research, policy

Aligning public health home visiting services with healthcare and social services can better address the goals and needs of the people and communities they serve. Literature in the early childcare service setting suggests that better coordination is associated with better quality outcomes, client engagement, and client retention, which is necessary for interventions to have their intended effects.^23^, ^38^, ^39^, ^40^ Cross‐sector collaboration in home visiting can be improved to better address family needs, especially among those experiencing the greatest social and economic adversities. This could be achieved through aligning services and systems by improving the domains of relational coordination (i.e., shared goals, shared knowledge, mutual respect) via the distribution of program marketing materials, ongoing communications, and cross‐pollination at meetings, as well as those of structural integration (i.e., shared physical space and data systems) by encouraging the adoption of memoranda of understanding to allow for badge and medical records access. This study adds to the literature on cross‐sector collaboration within the context of community‐based interventions and serves as a measurement guide to assess their collaborative activities with providers across sectors. Future research aims to compare collaboration measures to maternal‐child health outcomes to assess NFP program's impact on families experiencing adversities.

## Supporting information


**Appendix S1.** Collaboration survey measures and domains.
