# Supplementary material for: Changes in cross‐sector collaboration between nurse home visitors and community providers in the United States: A panel survey analysis
Source: Health Serv Res. 2023 Sep 28;59(Suppl 1):e14242. doi: 10.1111/1475-6773.14242 (PMC10796279; doi:10.1111/1475-6773.14242)
Supplement: Supplementary file 1 — Appendix S1. Collaboration survey measures and domains. [file HESR-59-0-s001.docx]

**Appendix**. Collaboration Survey Measures And Domains

| **Survey domain** | **Source of survey item(s)** | **Survey item(s)** | **Response options** |
| --- | --- | --- | --- |
| High quality communication:  1. Frequency  2. Timeliness  3. Accuracy  4. Problem-solving | Relational Coordination Scale | 1. When there is a need, how frequently do people in the following groups communicate with you about providing care and services to NFP clients?  2. When there is a need, do they communicate with you in a timely way about providing care and services to NFP clients?  3. When there is a need, do they communicate with you accurately about providing care and services to NFP clients?  4. When there is a problem with providing care and services to NFP clients, do people from these groups work with you to solve the problem? | 1. Never 2. Rarely 3. Occasionally 4. Often 5. Constantly |
| High quality relationships:  1. Shared goals  2. Shared knowledge  3. Mutual respect | Relational Coordination Scale | 1. Do people from the following groups share your goals for providing care and services to NFP clients?  2. Do they know about the work you do to provide care and services to NFP clients?  3. Do they respect the work you do to provide care and services for NFP clients? | 1. Not at all/Nothing 2. A little/Little 3. Somewhat/Some 4. A lot 5. Completely/Everything |
| Structural Integration  1. Shared space  2. Shared data  3. Shared policies  4. Shared funding | Adapted from  Interagency  Collaborative Activities  Scale^a^ | To what extent does your organization share the following resources  with your local [provider type] in:  1. Facility space  2. Record keeping and management information system data  3. Written agreements  4. Funding | 1- Not at all  2- Little  3- Somewhat  4- Considerable   1. Very much |
| ^a^Adapted from Greenbaum PE, Dedrick RF. Interagency Collaboration Activities Scale (IACAS). The Research and Training Center for Children’s Mental Health, University of South Florida. | | | |
